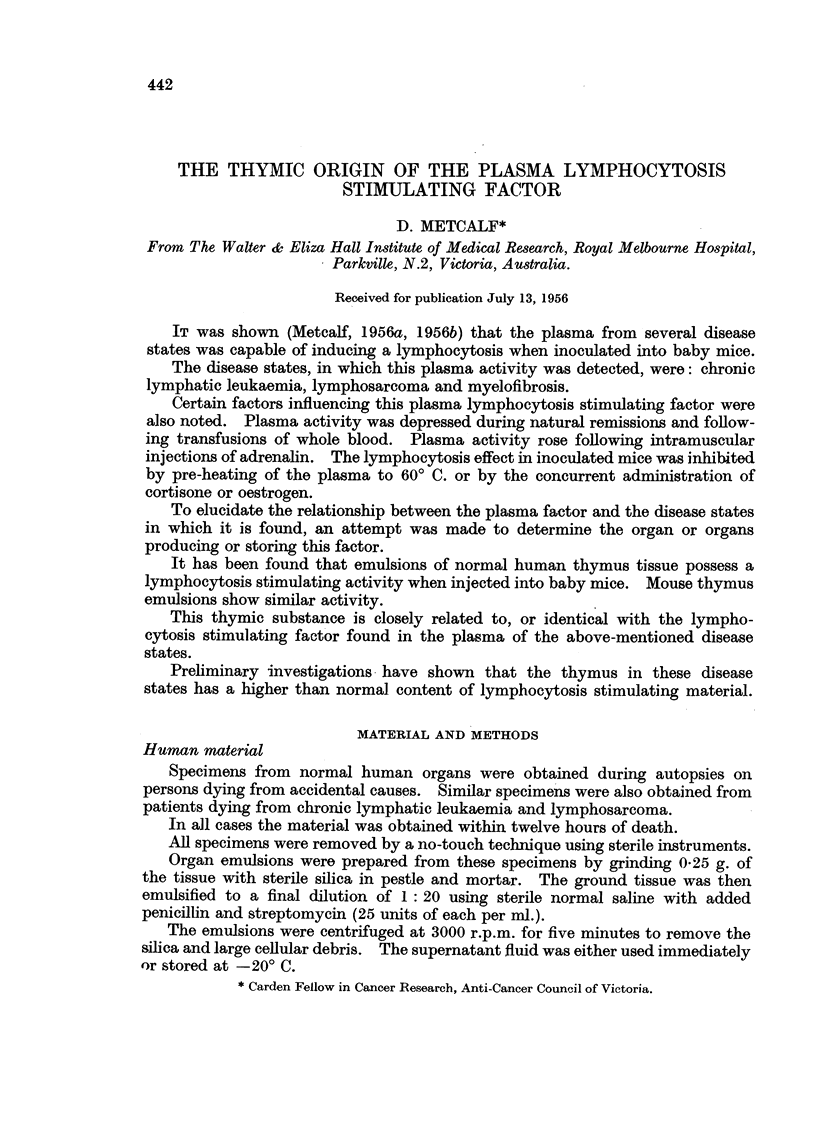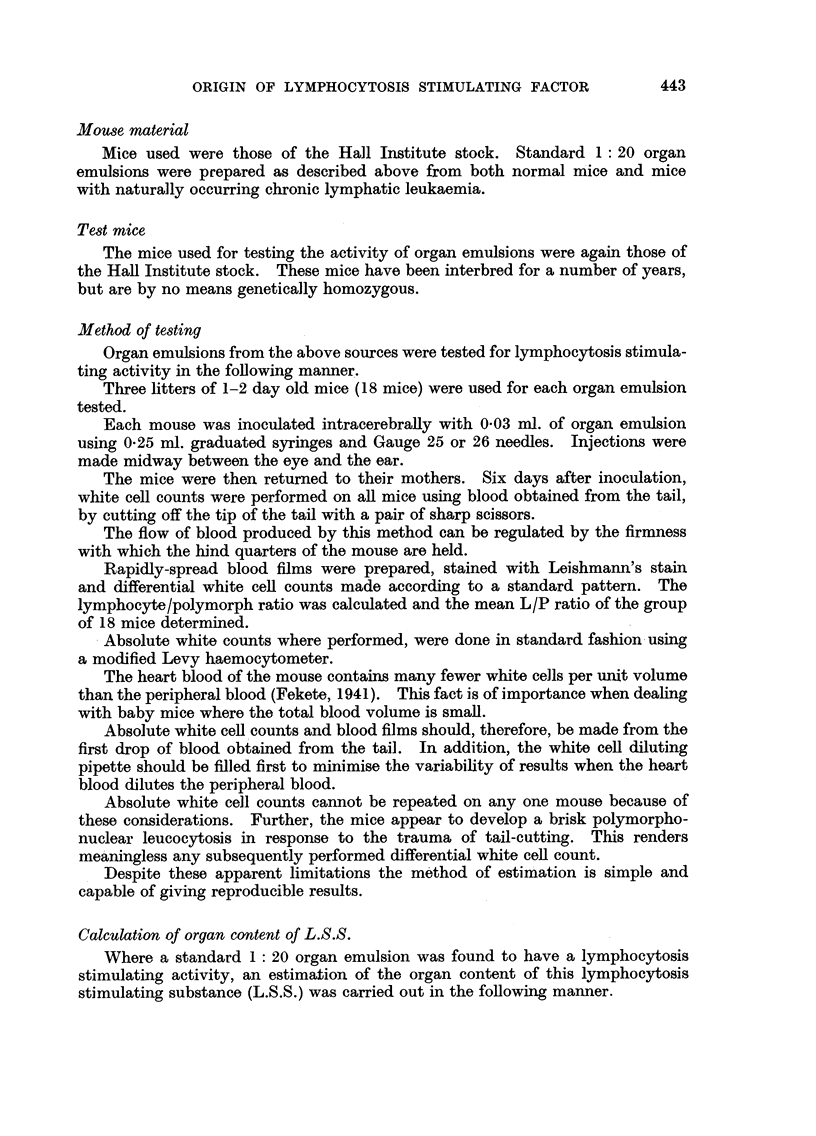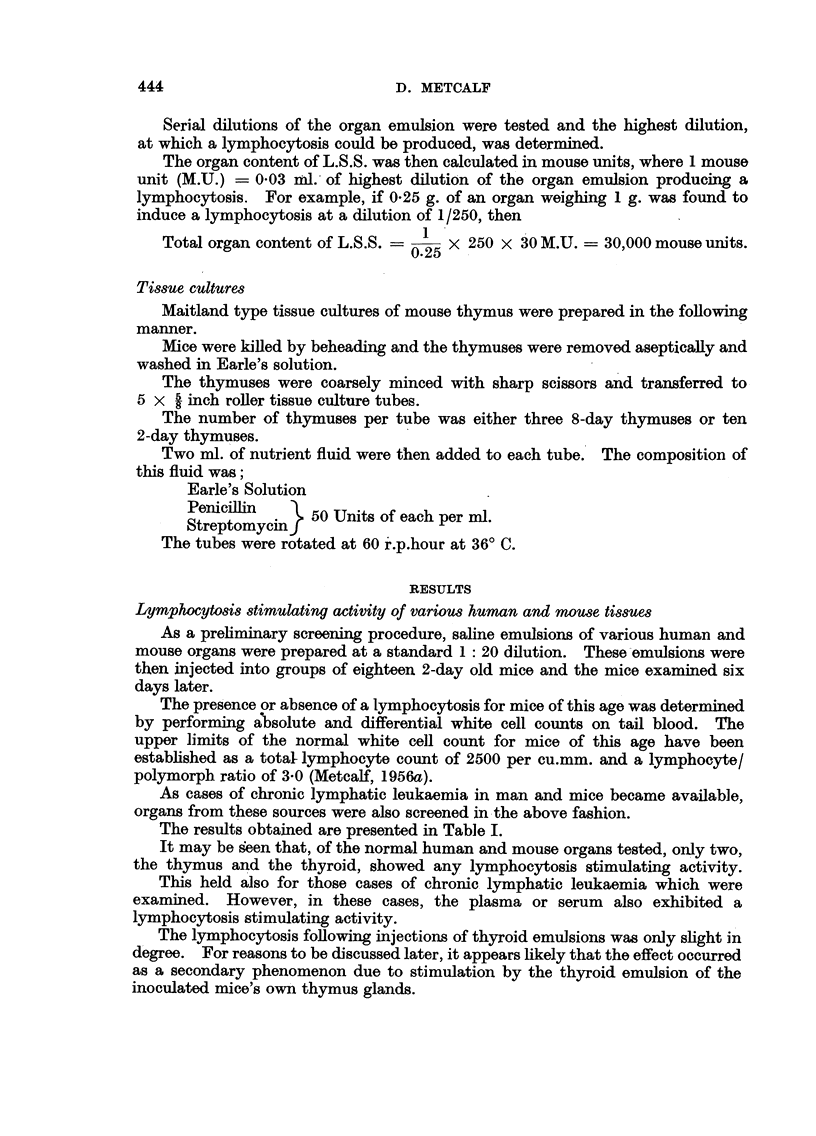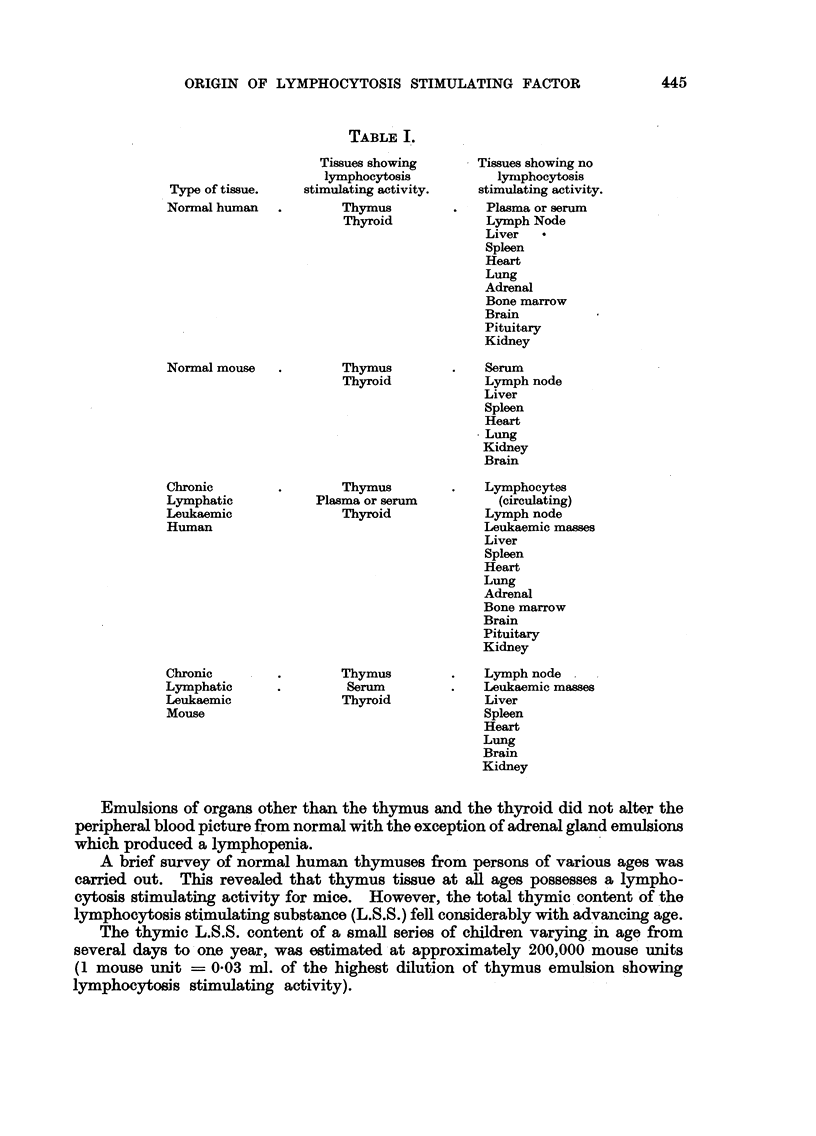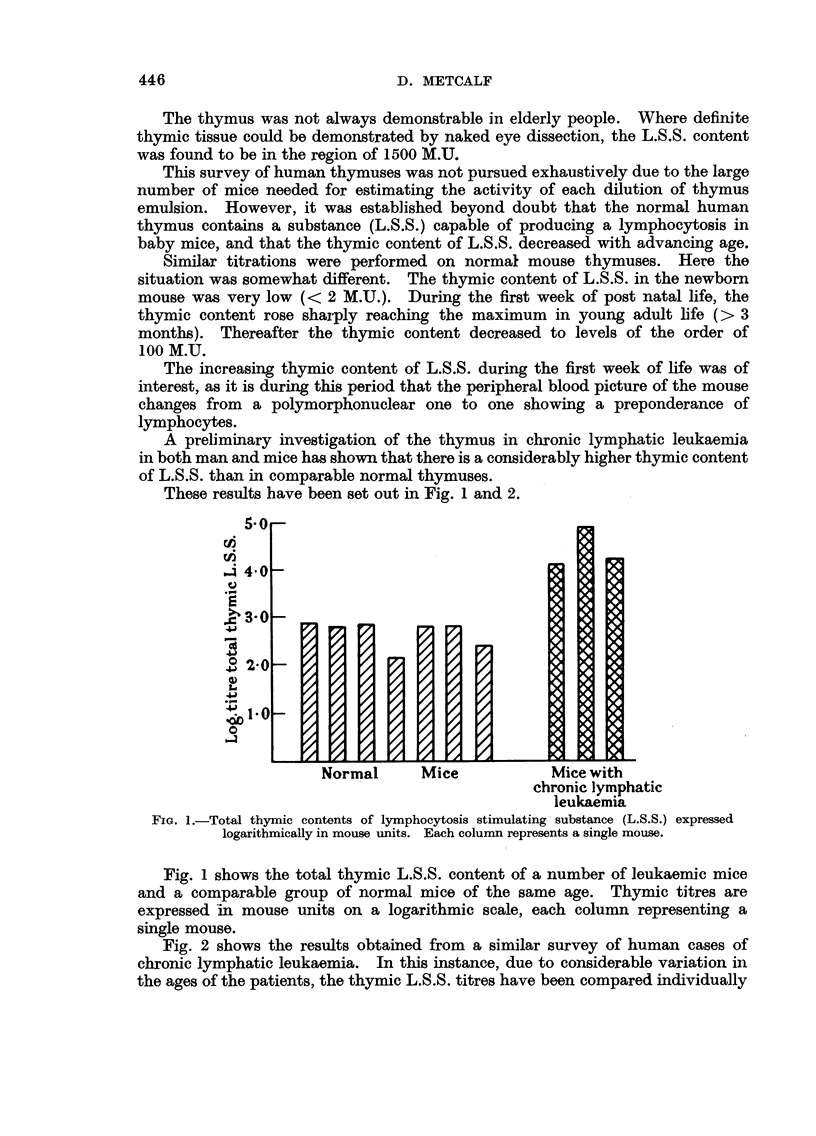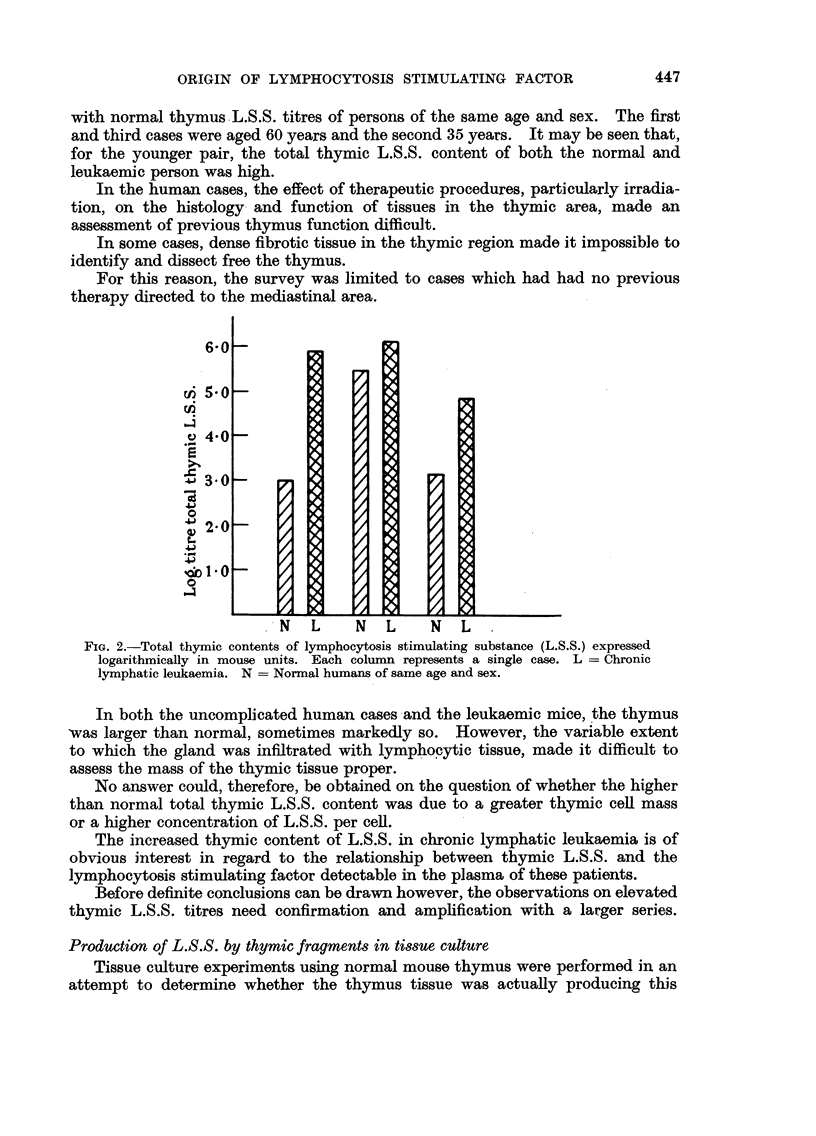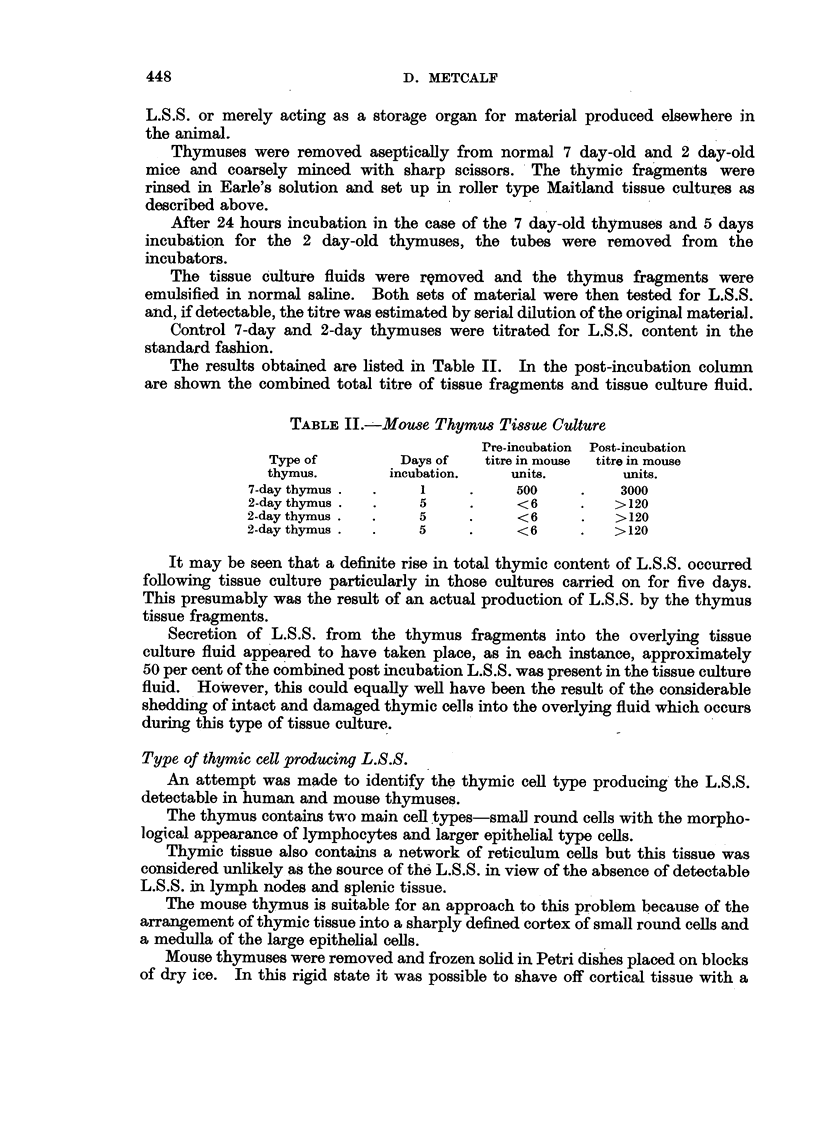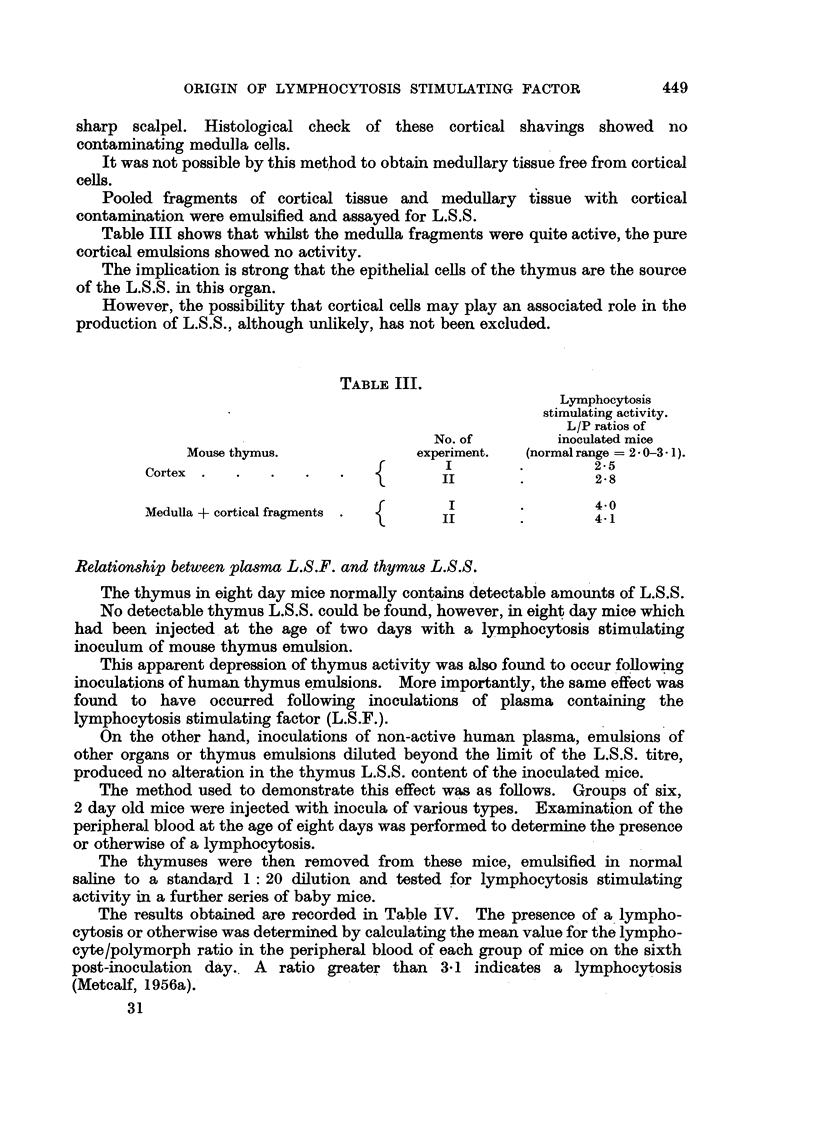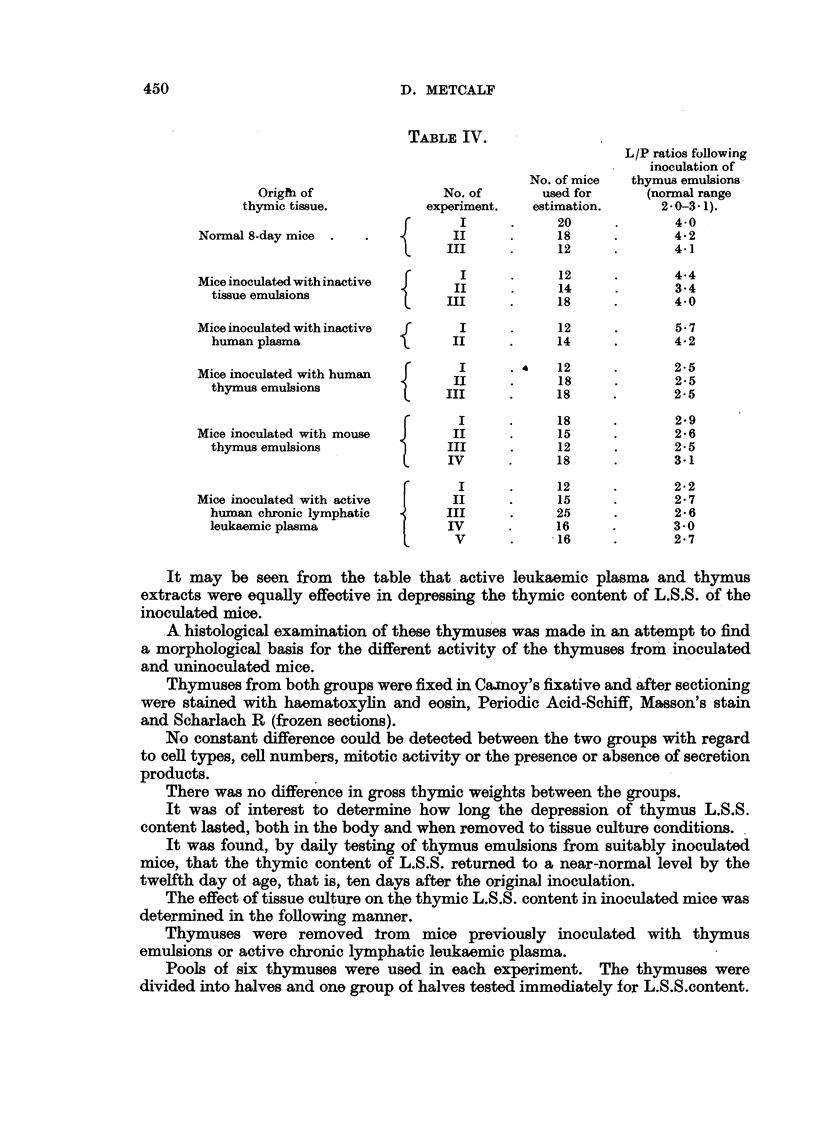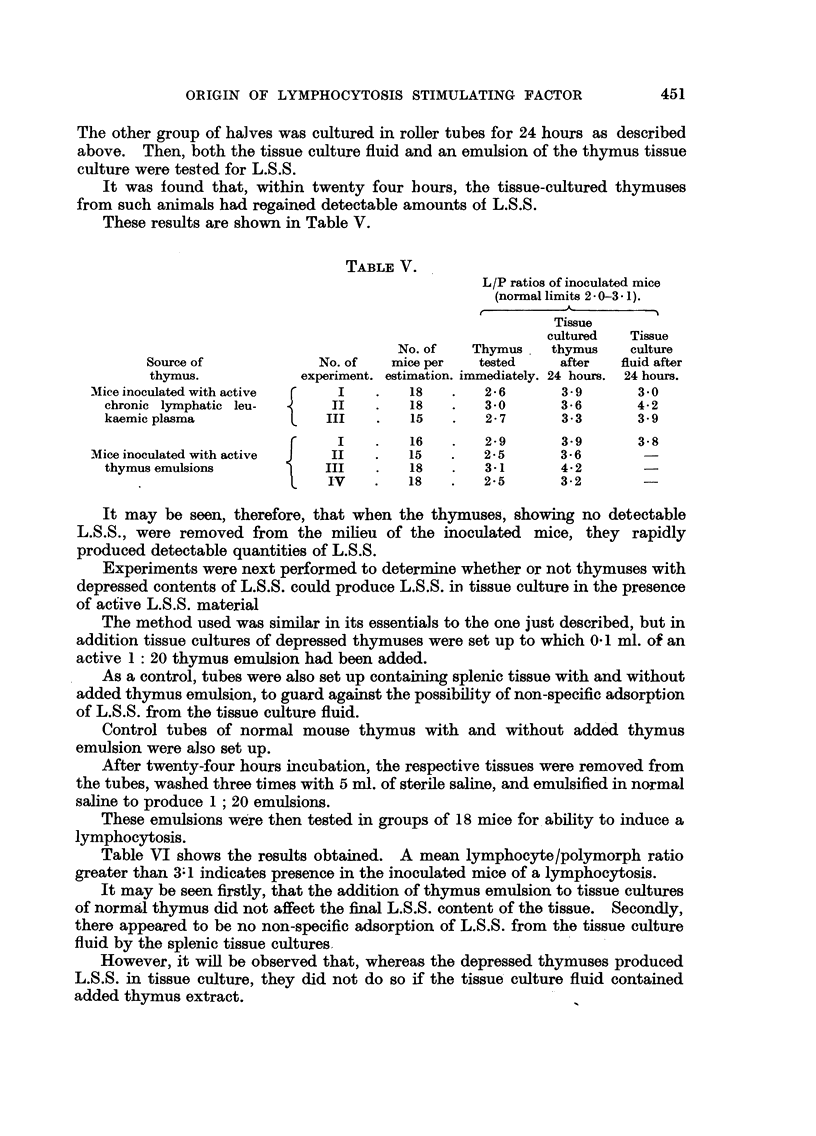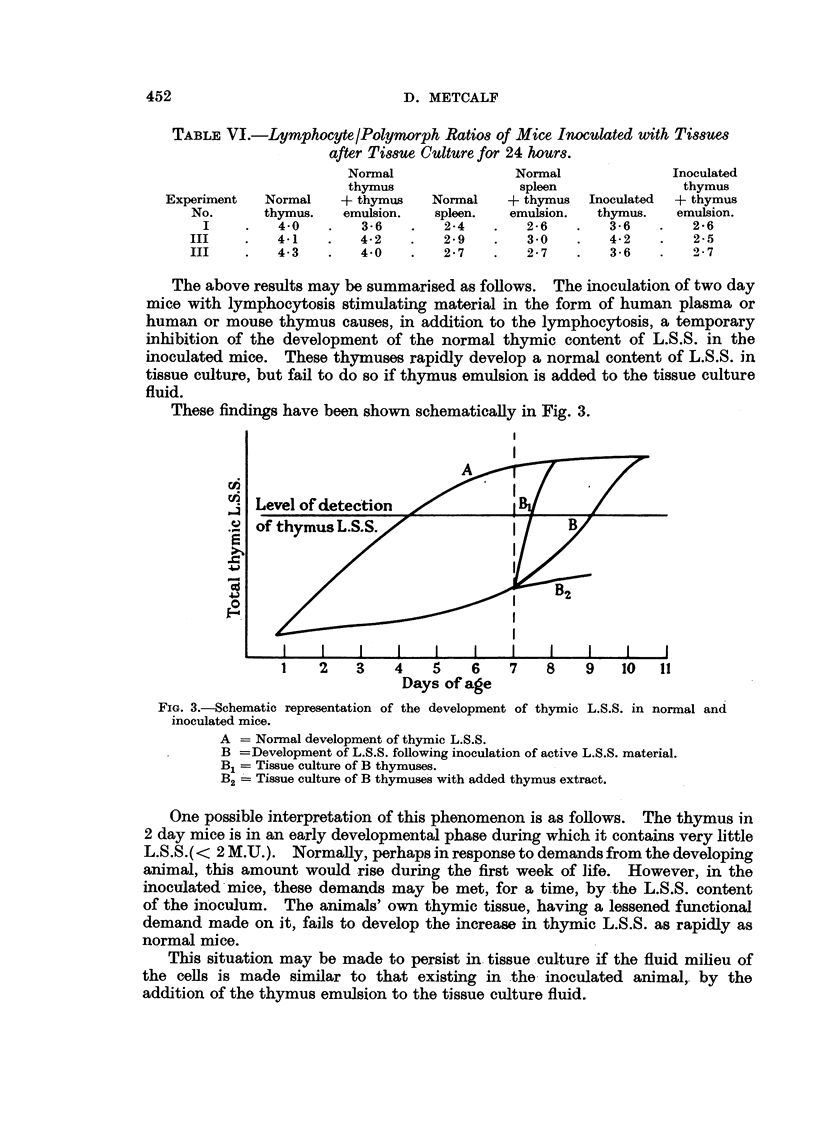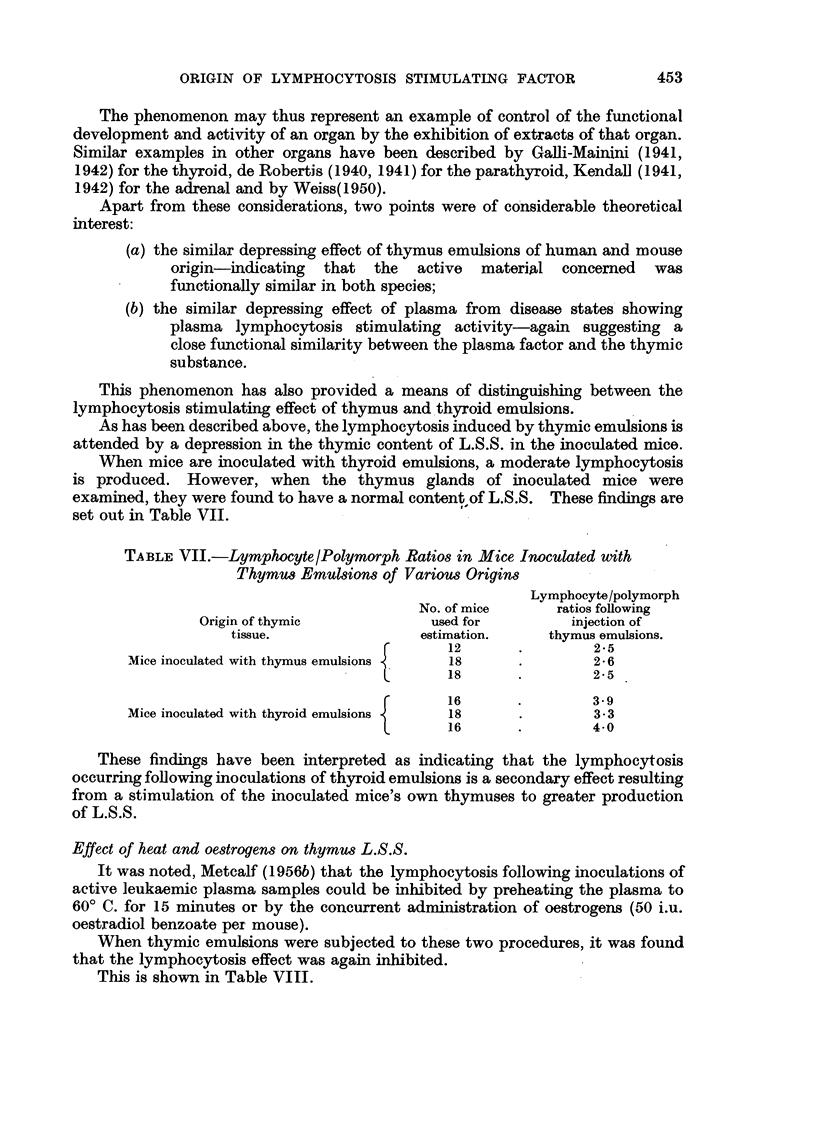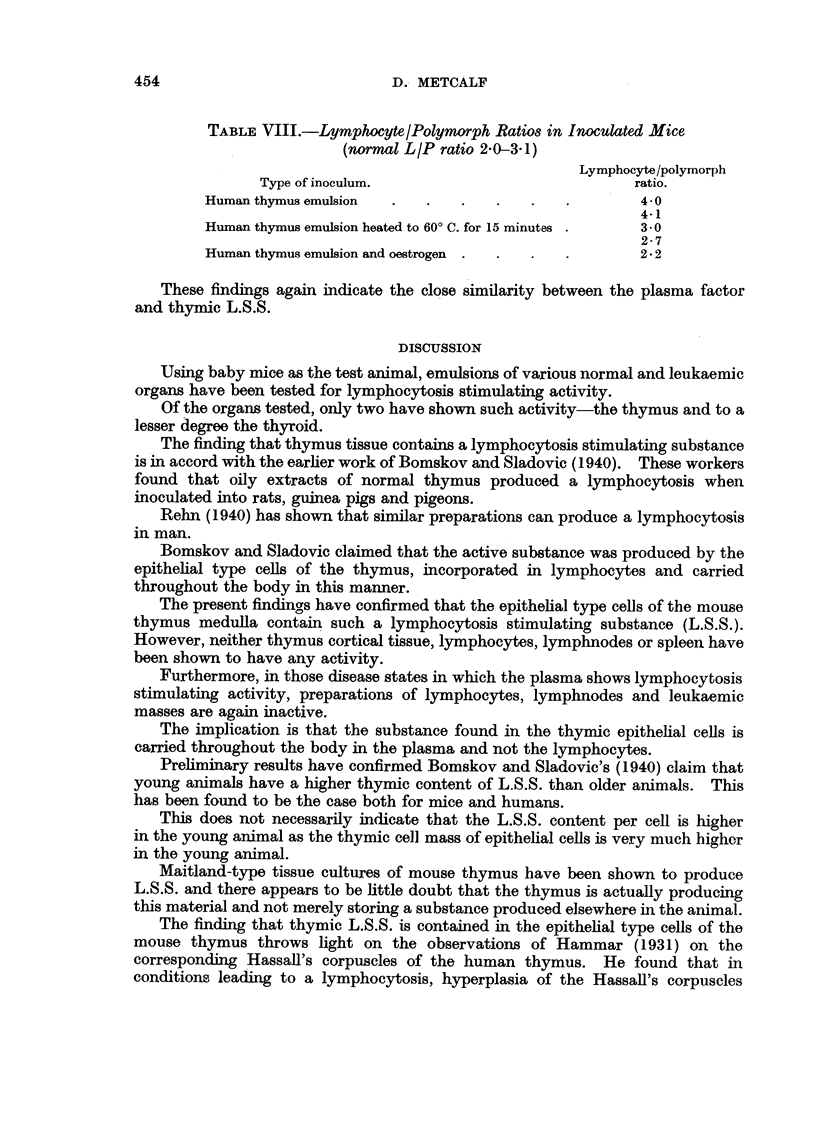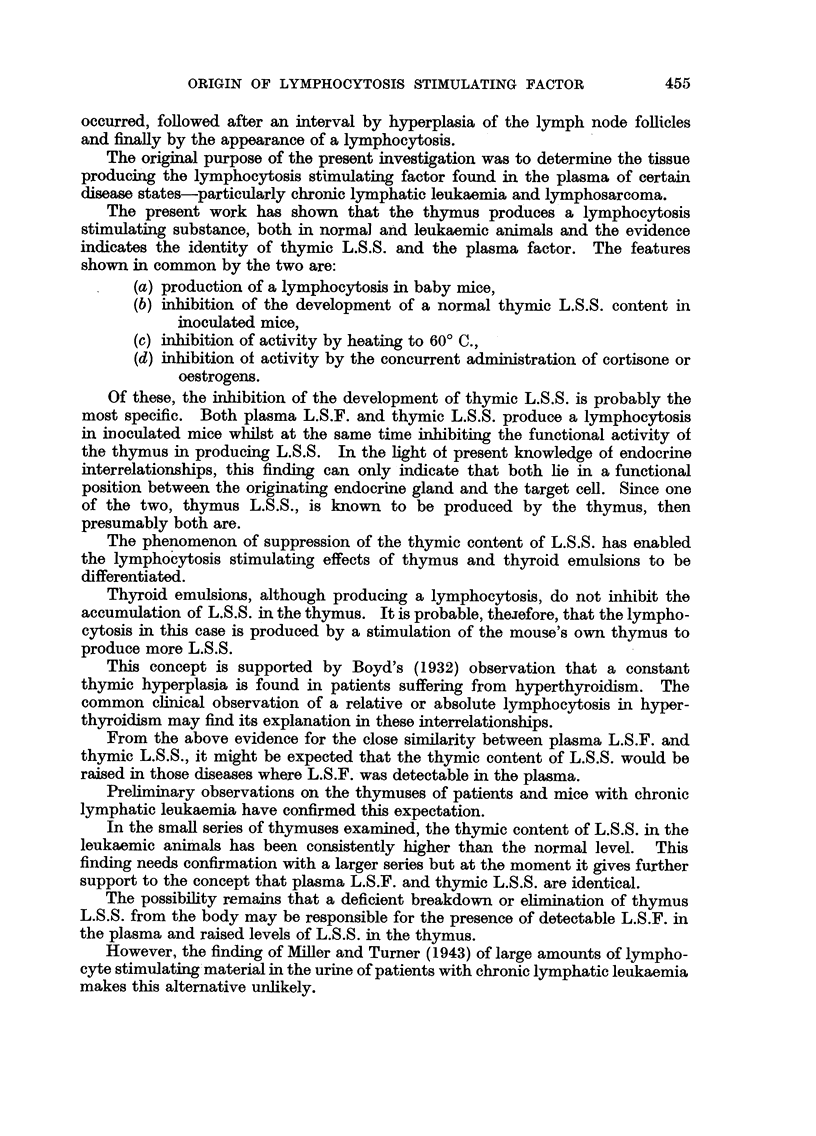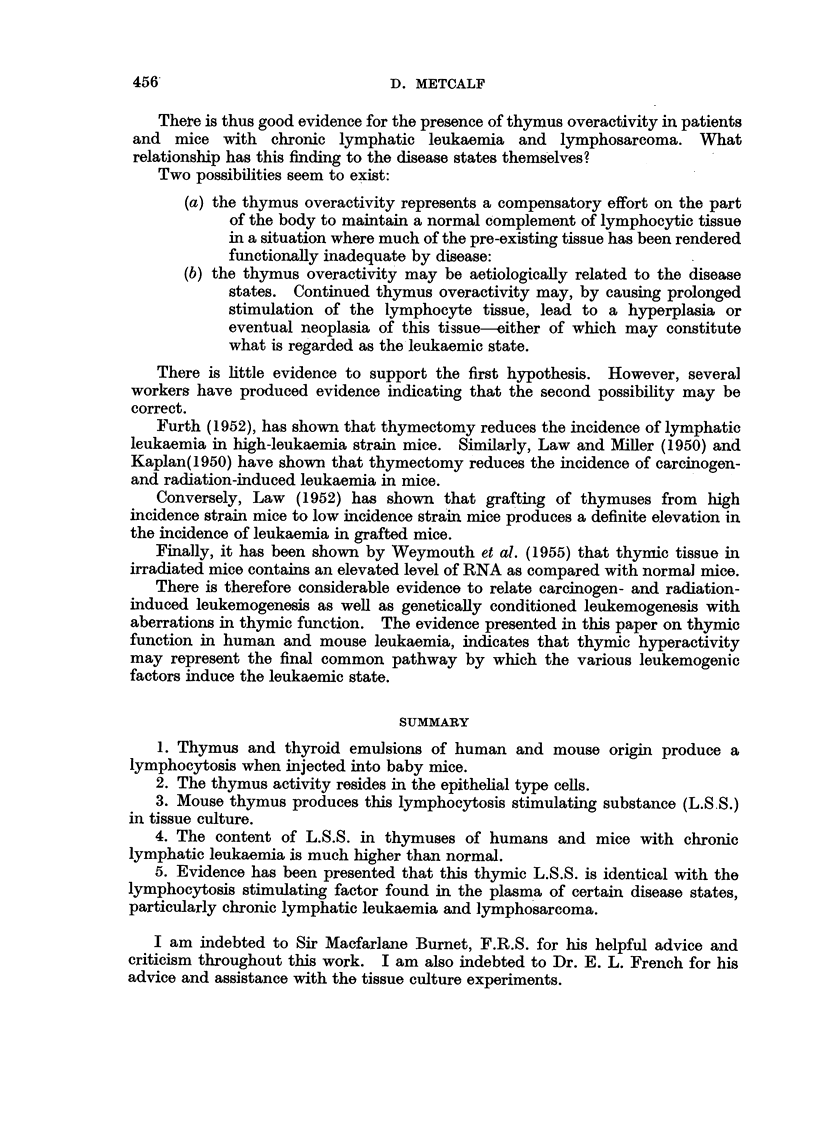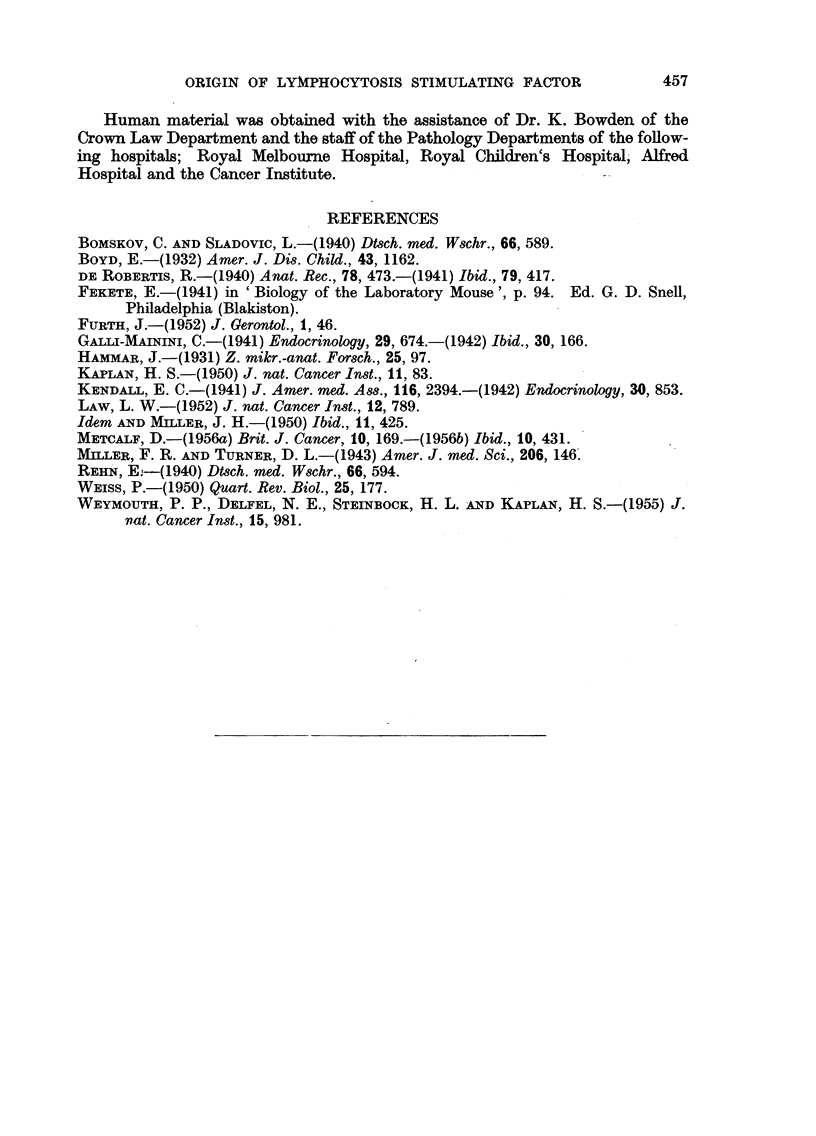# The Thymic Origin of the Plasma Lymphocytosis Stimulating Factor

**DOI:** 10.1038/bjc.1956.51

**Published:** 1956-09

**Authors:** D. Metcalf


### THE THYMIC ORIGIN OF THE PLASMA LYMPHOCYTOSIS STIMULATING FACTOR

D. METCALF*

*From The Walter & Eliza Hall Institute of Medical Research, Royal Melbourne Hospital, Parkville, N.2, Victoria, Australia.*

Received for publication July 13, 1956

IT was shown (Metcalf, 1956*a*, 1956*b*) that the plasma from several disease states was capable of inducing a lymphocytosis when inoculated into baby mice.

The disease states, in which this plasma activity was detected, were: chronic lymphatic leukaemia, lymphosarcoma and myelofibrosis.

Certain factors influencing this plasma lymphocytosis stimulating factor were also noted. Plasma activity was depressed during natural remissions and following transfusions of whole blood. Plasma activity rose following intramuscular injections of adrenalin. The lymphocytosis effect in inoculated mice was inhibited by pre-heating of the plasma to 60° C. or by the concurrent administration of cortisone or oestrogen.

To elucidate the relationship between the plasma factor and the disease states in which it is found, an attempt was made to determine the organ or organs producing or storing this factor.

It has been found that emulsions of normal human thymus tissue possess a lymphocytosis stimulating activity when injected into baby mice. Mouse thymus emulsions show similar activity.

This thymic substance is closely related to, or identical with the lymphocytosis stimulating factor found in the plasma of the above-mentioned disease states.

Preliminary investigations have shown that the thymus in these disease states has a higher than normal content of lymphocytosis stimulating material.

### MATERIAL AND METHODS

### *Human material*

Specimens from normal human organs were obtained during autopsies on persons dying from accidental causes. Similar specimens were also obtained from patients dying from chronic lymphatic leukaemia and lymphosarcoma.

In all cases the material was obtained within twelve hours of death.

All specimens were removed by a no-touch technique using sterile instruments.

Organ emulsions were prepared from these specimens by grinding 0·25 g. of the tissue with sterile silica in pestle and mortar. The ground tissue was then emulsified to a final dilution of 1 : 20 using sterile normal saline with added penicillin and streptomycin (25 units of each per ml.).

The emulsions were centrifuged at 3000 r.p.m. for five minutes to remove the silica and large cellular debris. The supernatant fluid was either used immediately or stored at −20° C.

* Carden Fellow in Cancer Research, Anti-Cancer Council of Victoria.

*Mouse material*

Mice used were those of the Hall Institute stock. Standard 1 : 20 organ emulsions were prepared as described above from both normal mice and mice with naturally occurring chronic lymphatic leukaemia.

*Test mice*

The mice used for testing the activity of organ emulsions were again those of the Hall Institute stock. These mice have been interbred for a number of years, but are by no means genetically homozygous.

*Method of testing*

Organ emulsions from the above sources were tested for lymphocytosis stimulating activity in the following manner.

Three litters of 1–2 day old mice (18 mice) were used for each organ emulsion tested.

Each mouse was inoculated intracerebrally with 0·03 ml. of organ emulsion using 0·25 ml. graduated syringes and Gauge 25 or 26 needles. Injections were made midway between the eye and the ear.

The mice were then returned to their mothers. Six days after inoculation, white cell counts were performed on all mice using blood obtained from the tail, by cutting off the tip of the tail with a pair of sharp scissors.

The flow of blood produced by this method can be regulated by the firmness with which the hind quarters of the mouse are held.

Rapidly-spread blood films were prepared, stained with Leishmann's stain and differential white cell counts made according to a standard pattern. The lymphocyte/polymorph ratio was calculated and the mean L/P ratio of the group of 18 mice determined.

Absolute white counts where performed, were done in standard fashion using a modified Levy haemocytometer.

The heart blood of the mouse contains many fewer white cells per unit volume than the peripheral blood (Fekete, 1941). This fact is of importance when dealing with baby mice where the total blood volume is small.

Absolute white cell counts and blood films should, therefore, be made from the first drop of blood obtained from the tail. In addition, the white cell diluting pipette should be filled first to minimise the variability of results when the heart blood dilutes the peripheral blood.

Absolute white cell counts cannot be repeated on any one mouse because of these considerations. Further, the mice appear to develop a brisk polymorphonuclear leucocytosis in response to the trauma of tail-cutting. This renders meaningless any subsequently performed differential white cell count.

Despite these apparent limitations the method of estimation is simple and capable of giving reproducible results.

*Calculation of organ content of L.S.S.*

Where a standard 1 : 20 organ emulsion was found to have a lymphocytosis stimulating activity, an estimation of the organ content of this lymphocytosis stimulating substance (L.S.S.) was carried out in the following manner.

Serial dilutions of the organ emulsion were tested and the highest dilution, at which a lymphocytosis could be produced, was determined.

The organ content of L.S.S. was then calculated in mouse units, where 1 mouse unit (M.U.) = 0·03 ml. of highest dilution of the organ emulsion producing a lymphocytosis. For example, if 0·25 g. of an organ weighing 1 g. was found to induce a lymphocytosis at a dilution of 1/250, then

$$\text{Total organ content of L.S.S.} = \frac{1}{0{\cdot}25} \times 250 \times 30\,\text{M.U.} = 30{,}000 \text{ mouse units.}$$

*Tissue cultures*

Maitland type tissue cultures of mouse thymus were prepared in the following manner.

Mice were killed by beheading and the thymuses were removed aseptically and washed in Earle's solution.

The thymuses were coarsely minced with sharp scissors and transferred to $5 \times \frac{5}{8}$ inch roller tissue culture tubes.

The number of thymuses per tube was either three 8-day thymuses or ten 2-day thymuses.

Two ml. of nutrient fluid were then added to each tube. The composition of this fluid was;

Earle's Solution
Penicillin, Streptomycin — 50 Units of each per ml.

The tubes were rotated at 60 r.p.hour at 36° C.

### RESULTS

*Lymphocytosis stimulating activity of various human and mouse tissues*

As a preliminary screening procedure, saline emulsions of various human and mouse organs were prepared at a standard 1 : 20 dilution. These emulsions were then injected into groups of eighteen 2-day old mice and the mice examined six days later.

The presence or absence of a lymphocytosis for mice of this age was determined by performing absolute and differential white cell counts on tail blood. The upper limits of the normal white cell count for mice of this age have been established as a total lymphocyte count of 2500 per cu.mm. and a lymphocyte/polymorph ratio of 3·0 (Metcalf, 1956*a*).

As cases of chronic lymphatic leukaemia in man and mice became available, organs from these sources were also screened in the above fashion.

The results obtained are presented in Table I.

It may be seen that, of the normal human and mouse organs tested, only two, the thymus and the thyroid, showed any lymphocytosis stimulating activity.

This held also for those cases of chronic lymphatic leukaemia which were examined. However, in these cases, the plasma or serum also exhibited a lymphocytosis stimulating activity.

The lymphocytosis following injections of thyroid emulsions was only slight in degree. For reasons to be discussed later, it appears likely that the effect occurred as a secondary phenomenon due to stimulation by the thyroid emulsion of the inoculated mice's own thymus glands.

TABLE I.

| Type of tissue. | Tissues showing lymphocytosis stimulating activity. | Tissues showing no lymphocytosis stimulating activity. |
|---|---|---|
| Normal human | Thymus<br>Thyroid | Plasma or serum<br>Lymph Node<br>Liver<br>Spleen<br>Heart<br>Lung<br>Adrenal<br>Bone marrow<br>Brain<br>Pituitary<br>Kidney |
| Normal mouse | Thymus<br>Thyroid | Serum<br>Lymph node<br>Liver<br>Spleen<br>Heart<br>Lung<br>Kidney<br>Brain |
| Chronic Lymphatic Leukaemic Human | Thymus<br>Plasma or serum<br>Thyroid | Lymphocytes (circulating)<br>Lymph node<br>Leukaemic masses<br>Liver<br>Spleen<br>Heart<br>Lung<br>Adrenal<br>Bone marrow<br>Brain<br>Pituitary<br>Kidney |
| Chronic Lymphatic Leukaemic Mouse | Thymus<br>Serum<br>Thyroid | Lymph node<br>Leukaemic masses<br>Liver<br>Spleen<br>Heart<br>Lung<br>Brain<br>Kidney |

Emulsions of organs other than the thymus and the thyroid did not alter the peripheral blood picture from normal with the exception of adrenal gland emulsions which produced a lymphopenia.

A brief survey of normal human thymuses from persons of various ages was carried out. This revealed that thymus tissue at all ages possesses a lymphocytosis stimulating activity for mice. However, the total thymic content of the lymphocytosis stimulating substance (L.S.S.) fell considerably with advancing age.

The thymic L.S.S. content of a small series of children varying in age from several days to one year, was estimated at approximately 200,000 mouse units (1 mouse unit = 0·03 ml. of the highest dilution of thymus emulsion showing lymphocytosis stimulating activity).

The thymus was not always demonstrable in elderly people. Where definite thymic tissue could be demonstrated by naked eye dissection, the L.S.S. content was found to be in the region of 1500 M.U.

This survey of human thymuses was not pursued exhaustively due to the large number of mice needed for estimating the activity of each dilution of thymus emulsion. However, it was established beyond doubt that the normal human thymus contains a substance (L.S.S.) capable of producing a lymphocytosis in baby mice, and that the thymic content of L.S.S. decreased with advancing age.

Similar titrations were performed on normal mouse thymuses. Here the situation was somewhat different. The thymic content of L.S.S. in the newborn mouse was very low ($< 2$ M.U.). During the first week of post natal life, the thymic content rose sharply reaching the maximum in young adult life ($> 3$ months). Thereafter the thymic content decreased to levels of the order of 100 M.U.

The increasing thymic content of L.S.S. during the first week of life was of interest, as it is during this period that the peripheral blood picture of the mouse changes from a polymorphonuclear one to one showing a preponderance of lymphocytes.

A preliminary investigation of the thymus in chronic lymphatic leukaemia in both man and mice has shown that there is a considerably higher thymic content of L.S.S. than in comparable normal thymuses.

These results have been set out in Fig. 1 and 2.

FIG. 1.—Total thymic contents of lymphocytosis stimulating substance (L.S.S.) expressed logarithmically in mouse units. Each column represents a single mouse.

Fig. 1 shows the total thymic L.S.S. content of a number of leukaemic mice and a comparable group of normal mice of the same age. Thymic titres are expressed in mouse units on a logarithmic scale, each column representing a single mouse.

Fig. 2 shows the results obtained from a similar survey of human cases of chronic lymphatic leukaemia. In this instance, due to considerable variation in the ages of the patients, the thymic L.S.S. titres have been compared individually with normal thymus L.S.S. titres of persons of the same age and sex. The first and third cases were aged 60 years and the second 35 years. It may be seen that, for the younger pair, the total thymic L.S.S. content of both the normal and leukaemic person was high.

In the human cases, the effect of therapeutic procedures, particularly irradiation, on the histology and function of tissues in the thymic area, made an assessment of previous thymus function difficult.

In some cases, dense fibrotic tissue in the thymic region made it impossible to identify and dissect free the thymus.

For this reason, the survey was limited to cases which had had no previous therapy directed to the mediastinal area.

FIG. 2.—Total thymic contents of lymphocytosis stimulating substance (L.S.S.) expressed logarithmically in mouse units. Each column represents a single case. L = Chronic lymphatic leukaemia. N = Normal humans of same age and sex.

In both the uncomplicated human cases and the leukaemic mice, the thymus was larger than normal, sometimes markedly so. However, the variable extent to which the gland was infiltrated with lymphocytic tissue, made it difficult to assess the mass of the thymic tissue proper.

No answer could, therefore, be obtained on the question of whether the higher than normal total thymic L.S.S. content was due to a greater thymic cell mass or a higher concentration of L.S.S. per cell.

The increased thymic content of L.S.S. in chronic lymphatic leukaemia is of obvious interest in regard to the relationship between thymic L.S.S. and the lymphocytosis stimulating factor detectable in the plasma of these patients.

Before definite conclusions can be drawn however, the observations on elevated thymic L.S.S. titres need confirmation and amplification with a larger series.

*Production of L.S.S. by thymic fragments in tissue culture*

Tissue culture experiments using normal mouse thymus were performed in an attempt to determine whether the thymus tissue was actually producing this

L.S.S. or merely acting as a storage organ for material produced elsewhere in the animal.

Thymuses were removed aseptically from normal 7 day-old and 2 day-old mice and coarsely minced with sharp scissors. The thymic fragments were rinsed in Earle's solution and set up in roller type Maitland tissue cultures as described above.

After 24 hours incubation in the case of the 7 day-old thymuses and 5 days incubation for the 2 day-old thymuses, the tubes were removed from the incubators.

The tissue culture fluids were removed and the thymus fragments were emulsified in normal saline. Both sets of material were then tested for L.S.S. and, if detectable, the titre was estimated by serial dilution of the original material.

Control 7-day and 2-day thymuses were titrated for L.S.S. content in the standard fashion.

The results obtained are listed in Table II. In the post-incubation column are shown the combined total titre of tissue fragments and tissue culture fluid.

TABLE II.—*Mouse Thymus Tissue Culture*

| Type of thymus. | Days of incubation. | Pre-incubation titre in mouse units. | Post-incubation titre in mouse units. |
|---|---|---|---|
| 7-day thymus | 1 | 500 | 3000 |
| 2-day thymus | 5 | $<6$ | $>120$ |
| 2-day thymus | 5 | $<6$ | $>120$ |
| 2-day thymus | 5 | $<6$ | $>120$ |

It may be seen that a definite rise in total thymic content of L.S.S. occurred following tissue culture particularly in those cultures carried on for five days. This presumably was the result of an actual production of L.S.S. by the thymus tissue fragments.

Secretion of L.S.S. from the thymus fragments into the overlying tissue culture fluid appeared to have taken place, as in each instance, approximately 50 per cent of the combined post incubation L.S.S. was present in the tissue culture fluid. However, this could equally well have been the result of the considerable shedding of intact and damaged thymic cells into the overlying fluid which occurs during this type of tissue culture.

*Type of thymic cell producing L.S.S.*

An attempt was made to identify the thymic cell type producing the L.S.S. detectable in human and mouse thymuses.

The thymus contains two main cell types—small round cells with the morphological appearance of lymphocytes and larger epithelial type cells.

Thymic tissue also contains a network of reticulum cells but this tissue was considered unlikely as the source of the L.S.S. in view of the absence of detectable L.S.S. in lymph nodes and splenic tissue.

The mouse thymus is suitable for an approach to this problem because of the arrangement of thymic tissue into a sharply defined cortex of small round cells and a medulla of the large epithelial cells.

Mouse thymuses were removed and frozen solid in Petri dishes placed on blocks of dry ice. In this rigid state it was possible to shave off cortical tissue with a sharp scalpel. Histological check of these cortical shavings showed no contaminating medulla cells.

It was not possible by this method to obtain medullary tissue free from cortical cells.

Pooled fragments of cortical tissue and medullary tissue with cortical contamination were emulsified and assayed for L.S.S.

Table III shows that whilst the medulla fragments were quite active, the pure cortical emulsions showed no activity.

The implication is strong that the epithelial cells of the thymus are the source of the L.S.S. in this organ.

However, the possibility that cortical cells may play an associated role in the production of L.S.S., although unlikely, has not been excluded.

TABLE III.

| Mouse thymus. | No. of experiment. | Lymphocytosis stimulating activity. L/P ratios of inoculated mice (normal range = 2·0–3·1). |
|---|---|---|
| Cortex | I | 2·5 |
| | II | 2·8 |
| Medulla + cortical fragments | I | 4·0 |
| | II | 4·1 |

*Relationship between plasma L.S.F. and thymus L.S.S.*

The thymus in eight day mice normally contains detectable amounts of L.S.S.

No detectable thymus L.S.S. could be found, however, in eight day mice which had been injected at the age of two days with a lymphocytosis stimulating inoculum of mouse thymus emulsion.

This apparent depression of thymus activity was also found to occur following inoculations of human thymus emulsions. More importantly, the same effect was found to have occurred following inoculations of plasma containing the lymphocytosis stimulating factor (L.S.F.).

On the other hand, inoculations of non-active human plasma, emulsions of other organs or thymus emulsions diluted beyond the limit of the L.S.S. titre, produced no alteration in the thymus L.S.S. content of the inoculated mice.

The method used to demonstrate this effect was as follows. Groups of six, 2 day old mice were injected with inocula of various types. Examination of the peripheral blood at the age of eight days was performed to determine the presence or otherwise of a lymphocytosis.

The thymuses were then removed from these mice, emulsified in normal saline to a standard 1 : 20 dilution and tested for lymphocytosis stimulating activity in a further series of baby mice.

The results obtained are recorded in Table IV. The presence of a lymphocytosis or otherwise was determined by calculating the mean value for the lymphocyte/polymorph ratio in the peripheral blood of each group of mice on the sixth post-inoculation day. A ratio greater than 3·1 indicates a lymphocytosis (Metcalf, 1956a).

TABLE IV.

| Origin of thymic tissue. | No. of experiment. | No. of mice used for estimation. | L/P ratios following inoculation of thymus emulsions (normal range 2·0–3·1). |
|---|---|---|---|
| Normal 8-day mice | I | 20 | 4·0 |
| | II | 18 | 4·2 |
| | III | 12 | 4·1 |
| Mice inoculated with inactive tissue emulsions | I | 12 | 4·4 |
| | II | 14 | 3·4 |
| | III | 18 | 4·0 |
| Mice inoculated with inactive human plasma | I | 12 | 5·7 |
| | II | 14 | 4·2 |
| Mice inoculated with human thymus emulsions | I | 12 | 2·5 |
| | II | 18 | 2·5 |
| | III | 18 | 2·5 |
| Mice inoculated with mouse thymus emulsions | I | 18 | 2·9 |
| | II | 15 | 2·6 |
| | III | 12 | 2·5 |
| | IV | 18 | 3·1 |
| Mice inoculated with active human chronic lymphatic leukaemic plasma | I | 12 | 2·2 |
| | II | 15 | 2·7 |
| | III | 25 | 2·6 |
| | IV | 16 | 3·0 |
| | V | 16 | 2·7 |

It may be seen from the table that active leukaemic plasma and thymus extracts were equally effective in depressing the thymic content of L.S.S. of the inoculated mice.

A histological examination of these thymuses was made in an attempt to find a morphological basis for the different activity of the thymuses from inoculated and uninoculated mice.

Thymuses from both groups were fixed in Carnoy's fixative and after sectioning were stained with haematoxylin and eosin, Periodic Acid-Schiff, Masson's stain and Scharlach R (frozen sections).

No constant difference could be detected between the two groups with regard to cell types, cell numbers, mitotic activity or the presence or absence of secretion products.

There was no difference in gross thymic weights between the groups.

It was of interest to determine how long the depression of thymus L.S.S. content lasted, both in the body and when removed to tissue culture conditions.

It was found, by daily testing of thymus emulsions from suitably inoculated mice, that the thymic content of L.S.S. returned to a near-normal level by the twelfth day of age, that is, ten days after the original inoculation.

The effect of tissue culture on the thymic L.S.S. content in inoculated mice was determined in the following manner.

Thymuses were removed from mice previously inoculated with thymus emulsions or active chronic lymphatic leukaemic plasma.

Pools of six thymuses were used in each experiment. The thymuses were divided into halves and one group of halves tested immediately for L.S.S.content.

The other group of halves was cultured in roller tubes for 24 hours as described above. Then, both the tissue culture fluid and an emulsion of the thymus tissue culture were tested for L.S.S.

It was found that, within twenty four hours, the tissue-cultured thymuses from such animals had regained detectable amounts of L.S.S.

These results are shown in Table V.

TABLE V.

| Source of thymus. | No. of experiment. | No. of mice per estimation. | L/P ratios of inoculated mice (normal limits 2·0–3·1). Thymus tested immediately. | Tissue cultured thymus after 24 hours. | Tissue culture fluid after 24 hours. |
|---|---|---|---|---|---|
| Mice inoculated with active chronic lymphatic leukaemic plasma | I | 18 | 2·6 | 3·9 | 3·0 |
| | II | 18 | 3·0 | 3·6 | 4·2 |
| | III | 15 | 2·7 | 3·3 | 3·9 |
| Mice inoculated with active thymus emulsions | I | 16 | 2·9 | 3·9 | 3·8 |
| | II | 15 | 2·5 | 3·6 | — |
| | III | 18 | 3·1 | 4·2 | — |
| | IV | 18 | 2·5 | 3·2 | — |

It may be seen, therefore, that when the thymuses, showing no detectable L.S.S., were removed from the milieu of the inoculated mice, they rapidly produced detectable quantities of L.S.S.

Experiments were next performed to determine whether or not thymuses with depressed contents of L.S.S. could produce L.S.S. in tissue culture in the presence of active L.S.S. material

The method used was similar in its essentials to the one just described, but in addition tissue cultures of depressed thymuses were set up to which 0·1 ml. of an active 1 : 20 thymus emulsion had been added.

As a control, tubes were also set up containing splenic tissue with and without added thymus emulsion, to guard against the possibility of non-specific adsorption of L.S.S. from the tissue culture fluid.

Control tubes of normal mouse thymus with and without added thymus emulsion were also set up.

After twenty-four hours incubation, the respective tissues were removed from the tubes, washed three times with 5 ml. of sterile saline, and emulsified in normal saline to produce 1 ; 20 emulsions.

These emulsions were then tested in groups of 18 mice for ability to induce a lymphocytosis.

Table VI shows the results obtained. A mean lymphocyte/polymorph ratio greater than 3·1 indicates presence in the inoculated mice of a lymphocytosis.

It may be seen firstly, that the addition of thymus emulsion to tissue cultures of normal thymus did not affect the final L.S.S. content of the tissue. Secondly, there appeared to be no non-specific adsorption of L.S.S. from the tissue culture fluid by the splenic tissue cultures.

However, it will be observed that, whereas the depressed thymuses produced L.S.S. in tissue culture, they did not do so if the tissue culture fluid contained added thymus extract.

TABLE VI.—*Lymphocyte/Polymorph Ratios of Mice Inoculated with Tissues after Tissue Culture for 24 hours.*

| Experiment No. | Normal thymus. | Normal thymus + thymus emulsion. | Normal spleen. | Normal spleen + thymus emulsion. | Inoculated thymus. | Inoculated thymus + thymus emulsion. |
|---|---|---|---|---|---|---|
| I | 4·0 | 3·6 | 2·4 | 2·6 | 3·6 | 2·6 |
| III | 4·1 | 4·2 | 2·9 | 3·0 | 4·2 | 2·5 |
| III | 4·3 | 4·0 | 2·7 | 2·7 | 3·6 | 2·7 |

The above results may be summarised as follows. The inoculation of two day mice with lymphocytosis stimulating material in the form of human plasma or human or mouse thymus causes, in addition to the lymphocytosis, a temporary inhibition of the development of the normal thymic content of L.S.S. in the inoculated mice. These thymuses rapidly develop a normal content of L.S.S. in tissue culture, but fail to do so if thymus emulsion is added to the tissue culture fluid.

These findings have been shown schematically in Fig. 3.

FIG. 3.—Schematic representation of the development of thymic L.S.S. in normal and inoculated mice.

A = Normal development of thymic L.S.S.
B = Development of L.S.S. following inoculation of active L.S.S. material.
$B_1$ = Tissue culture of B thymuses.
$B_2$ = Tissue culture of B thymuses with added thymus extract.

One possible interpretation of this phenomenon is as follows. The thymus in 2 day mice is in an early developmental phase during which it contains very little L.S.S.(< 2 M.U.). Normally, perhaps in response to demands from the developing animal, this amount would rise during the first week of life. However, in the inoculated mice, these demands may be met, for a time, by the L.S.S. content of the inoculum. The animals' own thymic tissue, having a lessened functional demand made on it, fails to develop the increase in thymic L.S.S. as rapidly as normal mice.

This situation may be made to persist in tissue culture if the fluid milieu of the cells is made similar to that existing in the inoculated animal, by the addition of the thymus emulsion to the tissue culture fluid.

The phenomenon may thus represent an example of control of the functional development and activity of an organ by the exhibition of extracts of that organ. Similar examples in other organs have been described by Galli-Mainini (1941, 1942) for the thyroid, de Robertis (1940, 1941) for the parathyroid, Kendall (1941, 1942) for the adrenal and by Weiss(1950).

Apart from these considerations, two points were of considerable theoretical interest:

(*a*) the similar depressing effect of thymus emulsions of human and mouse origin—indicating that the active material concerned was functionally similar in both species;

(*b*) the similar depressing effect of plasma from disease states showing plasma lymphocytosis stimulating activity—again suggesting a close functional similarity between the plasma factor and the thymic substance.

This phenomenon has also provided a means of distinguishing between the lymphocytosis stimulating effect of thymus and thyroid emulsions.

As has been described above, the lymphocytosis induced by thymic emulsions is attended by a depression in the thymic content of L.S.S. in the inoculated mice.

When mice are inoculated with thyroid emulsions, a moderate lymphocytosis is produced. However, when the thymus glands of inoculated mice were examined, they were found to have a normal content of L.S.S. These findings are set out in Table VII.

TABLE VII.—*Lymphocyte/Polymorph Ratios in Mice Inoculated with Thymus Emulsions of Various Origins*

| Origin of thymic tissue. | No. of mice used for estimation. | Lymphocyte/polymorph ratios following injection of thymus emulsions. |
|---|---|---|
| Mice inoculated with thymus emulsions | 12 | 2·5 |
| | 18 | 2·6 |
| | 18 | 2·5 |
| Mice inoculated with thyroid emulsions | 16 | 3·9 |
| | 18 | 3·3 |
| | 16 | 4·0 |

These findings have been interpreted as indicating that the lymphocytosis occurring following inoculations of thyroid emulsions is a secondary effect resulting from a stimulation of the inoculated mice's own thymuses to greater production of L.S.S.

*Effect of heat and oestrogens on thymus L.S.S.*

It was noted, Metcalf (1956*b*) that the lymphocytosis following inoculations of active leukaemic plasma samples could be inhibited by preheating the plasma to 60° C. for 15 minutes or by the concurrent administration of oestrogens (50 i.u. oestradiol benzoate per mouse).

When thymic emulsions were subjected to these two procedures, it was found that the lymphocytosis effect was again inhibited.

This is shown in Table VIII.

TABLE VIII.—*Lymphocyte/Polymorph Ratios in Inoculated Mice (normal L/P ratio* 2·0–3·1)

| Type of inoculum. | Lymphocyte/polymorph ratio. |
|---|---|
| Human thymus emulsion . . . . . . . | 4·0 |
| | 4·1 |
| Human thymus emulsion heated to 60° C. for 15 minutes . | 3·0 |
| | 2·7 |
| Human thymus emulsion and oestrogen . . . . | 2·2 |

These findings again indicate the close similarity between the plasma factor and thymic L.S.S.

DISCUSSION

Using baby mice as the test animal, emulsions of various normal and leukaemic organs have been tested for lymphocytosis stimulating activity.

Of the organs tested, only two have shown such activity—the thymus and to a lesser degree the thyroid.

The finding that thymus tissue contains a lymphocytosis stimulating substance is in accord with the earlier work of Bomskov and Sladovic (1940). These workers found that oily extracts of normal thymus produced a lymphocytosis when inoculated into rats, guinea pigs and pigeons.

Rehn (1940) has shown that similar preparations can produce a lymphocytosis in man.

Bomskov and Sladovic claimed that the active substance was produced by the epithelial type cells of the thymus, incorporated in lymphocytes and carried throughout the body in this manner.

The present findings have confirmed that the epithelial type cells of the mouse thymus medulla contain such a lymphocytosis stimulating substance (L.S.S.). However, neither thymus cortical tissue, lymphocytes, lymphnodes or spleen have been shown to have any activity.

Furthermore, in those disease states in which the plasma shows lymphocytosis stimulating activity, preparations of lymphocytes, lymphnodes and leukaemic masses are again inactive.

The implication is that the substance found in the thymic epithelial cells is carried throughout the body in the plasma and not the lymphocytes.

Preliminary results have confirmed Bomskov and Sladovic's (1940) claim that young animals have a higher thymic content of L.S.S. than older animals. This has been found to be the case both for mice and humans.

This does not necessarily indicate that the L.S.S. content per cell is higher in the young animal as the thymic cell mass of epithelial cells is very much higher in the young animal.

Maitland-type tissue cultures of mouse thymus have been shown to produce L.S.S. and there appears to be little doubt that the thymus is actually producing this material and not merely storing a substance produced elsewhere in the animal.

The finding that thymic L.S.S. is contained in the epithelial type cells of the mouse thymus throws light on the observations of Hammar (1931) on the corresponding Hassall's corpuscles of the human thymus. He found that in conditions leading to a lymphocytosis, hyperplasia of the Hassall's corpuscles occurred, followed after an interval by hyperplasia of the lymph node follicles and finally by the appearance of a lymphocytosis.

The original purpose of the present investigation was to determine the tissue producing the lymphocytosis stimulating factor found in the plasma of certain disease states—particularly chronic lymphatic leukaemia and lymphosarcoma.

The present work has shown that the thymus produces a lymphocytosis stimulating substance, both in normal and leukaemic animals and the evidence indicates the identity of thymic L.S.S. and the plasma factor. The features shown in common by the two are:

(*a*) production of a lymphocytosis in baby mice,
(*b*) inhibition of the development of a normal thymic L.S.S. content in inoculated mice,
(*c*) inhibition of activity by heating to 60° C.,
(*d*) inhibition of activity by the concurrent administration of cortisone or oestrogens.

Of these, the inhibition of the development of thymic L.S.S. is probably the most specific. Both plasma L.S.F. and thymic L.S.S. produce a lymphocytosis in inoculated mice whilst at the same time inhibiting the functional activity of the thymus in producing L.S.S. In the light of present knowledge of endocrine interrelationships, this finding can only indicate that both lie in a functional position between the originating endocrine gland and the target cell. Since one of the two, thymus L.S.S., is known to be produced by the thymus, then presumably both are.

The phenomenon of suppression of the thymic content of L.S.S. has enabled the lymphocytosis stimulating effects of thymus and thyroid emulsions to be differentiated.

Thyroid emulsions, although producing a lymphocytosis, do not inhibit the accumulation of L.S.S. in the thymus. It is probable, therefore, that the lymphocytosis in this case is produced by a stimulation of the mouse's own thymus to produce more L.S.S.

This concept is supported by Boyd's (1932) observation that a constant thymic hyperplasia is found in patients suffering from hyperthyroidism. The common clinical observation of a relative or absolute lymphocytosis in hyperthyroidism may find its explanation in these interrelationships.

From the above evidence for the close similarity between plasma L.S.F. and thymic L.S.S., it might be expected that the thymic content of L.S.S. would be raised in those diseases where L.S.F. was detectable in the plasma.

Preliminary observations on the thymuses of patients and mice with chronic lymphatic leukaemia have confirmed this expectation.

In the small series of thymuses examined, the thymic content of L.S.S. in the leukaemic animals has been consistently higher than the normal level. This finding needs confirmation with a larger series but at the moment it gives further support to the concept that plasma L.S.F. and thymic L.S.S. are identical.

The possibility remains that a deficient breakdown or elimination of thymus L.S.S. from the body may be responsible for the presence of detectable L.S.F. in the plasma and raised levels of L.S.S. in the thymus.

However, the finding of Miller and Turner (1943) of large amounts of lymphocyte stimulating material in the urine of patients with chronic lymphatic leukaemia makes this alternative unlikely.

There is thus good evidence for the presence of thymus overactivity in patients and mice with chronic lymphatic leukaemia and lymphosarcoma. What relationship has this finding to the disease states themselves?

Two possibilities seem to exist:

(*a*) the thymus overactivity represents a compensatory effort on the part of the body to maintain a normal complement of lymphocytic tissue in a situation where much of the pre-existing tissue has been rendered functionally inadequate by disease:

(*b*) the thymus overactivity may be aetiologically related to the disease states. Continued thymus overactivity may, by causing prolonged stimulation of the lymphocyte tissue, lead to a hyperplasia or eventual neoplasia of this tissue—either of which may constitute what is regarded as the leukaemic state.

There is little evidence to support the first hypothesis. However, several workers have produced evidence indicating that the second possibility may be correct.

Furth (1952), has shown that thymectomy reduces the incidence of lymphatic leukaemia in high-leukaemia strain mice. Similarly, Law and Miller (1950) and Kaplan(1950) have shown that thymectomy reduces the incidence of carcinogen- and radiation-induced leukaemia in mice.

Conversely, Law (1952) has shown that grafting of thymuses from high incidence strain mice to low incidence strain mice produces a definite elevation in the incidence of leukaemia in grafted mice.

Finally, it has been shown by Weymouth *et al.* (1955) that thymic tissue in irradiated mice contains an elevated level of RNA as compared with normal mice.

There is therefore considerable evidence to relate carcinogen- and radiation-induced leukemogenesis as well as genetically conditioned leukemogenesis with aberrations in thymic function. The evidence presented in this paper on thymic function in human and mouse leukaemia, indicates that thymic hyperactivity may represent the final common pathway by which the various leukemogenic factors induce the leukaemic state.

### SUMMARY

1. Thymus and thyroid emulsions of human and mouse origin produce a lymphocytosis when injected into baby mice.

2. The thymus activity resides in the epithelial type cells.

3. Mouse thymus produces this lymphocytosis stimulating substance (L.S.S.) in tissue culture.

4. The content of L.S.S. in thymuses of humans and mice with chronic lymphatic leukaemia is much higher than normal.

5. Evidence has been presented that this thymic L.S.S. is identical with the lymphocytosis stimulating factor found in the plasma of certain disease states, particularly chronic lymphatic leukaemia and lymphosarcoma.

I am indebted to Sir Macfarlane Burnet, F.R.S. for his helpful advice and criticism throughout this work. I am also indebted to Dr. E. L. French for his advice and assistance with the tissue culture experiments.

Human material was obtained with the assistance of Dr. K. Bowden of the Crown Law Department and the staff of the Pathology Departments of the following hospitals; Royal Melbourne Hospital, Royal Children's Hospital, Alfred Hospital and the Cancer Institute.

### REFERENCES

BOMSKOV, C. AND SLADOVIC, L.—(1940) *Dtsch. med. Wschr.*, **66**, 589.
BOYD, E.—(1932) *Amer. J. Dis. Child.*, **43**, 1162.
DE ROBERTIS, R.—(1940) *Anat. Rec.*, **78**, 473.—(1941) *Ibid.*, **79**, 417.
FEKETE, E.—(1941) in 'Biology of the Laboratory Mouse', p. 94. Ed. G. D. Snell, Philadelphia (Blakiston).
FURTH, J.—(1952) *J. Gerontol.*, **1**, 46.
GALLI-MAININI, C.—(1941) *Endocrinology*, **29**, 674.—(1942) *Ibid.*, **30**, 166.
HAMMAR, J.—(1931) *Z. mikr.-anat. Forsch.*, **25**, 97.
KAPLAN, H. S.—(1950) *J. nat. Cancer Inst.*, **11**, 83.
KENDALL, E. C.—(1941) *J. Amer. med. Ass.*, **116**, 2394.—(1942) *Endocrinology*, **30**, 853.
LAW, L. W.—(1952) *J. nat. Cancer Inst.*, **12**, 789.
*Idem* AND MILLER, J. H.—(1950) *Ibid.*, **11**, 425.
METCALF, D.—(1956*a*) *Brit. J. Cancer*, **10**, 169.—(1956*b*) *Ibid.*, **10**, 431.
MILLER, F. R. AND TURNER, D. L.—(1943) *Amer. J. med. Sci.*, **206**, 146.
REHN, E.—(1940) *Dtsch. med. Wschr.*, **66**, 594.
WEISS, P.—(1950) *Quart. Rev. Biol.*, **25**, 177.
WEYMOUTH, P. P., DELFEL, N. E., STEINBOCK, H. L. AND KAPLAN, H. S.—(1955) *J. nat. Cancer Inst.*, **15**, 981.